# Neuropharmacological properties of farnesol in Murine model

**Published:** 2016

**Authors:** M. Shahnouri, M. Abouhosseini Tabari, A. Araghi

**Affiliations:** 1 Faculty of Veterinary Medicine, Babol Branch, Islamic Azad University, Babol, Iran;; 2 Department of Pharmacology, Faculty of Veterinary Medicine, Amol University of Special Modern Technologies, Amol, Iran;; 3 Department of Clinical Pathology, Faculty of Veterinary Medicine, Amol University of Special Modern Technologies, Amol, Iran

**Keywords:** Anxiolytic, Anti-nociceptive, Cortisol, Depressive behavior, Farnesol

## Abstract

Research on new compounds of therapeutic value for behavioral disorders has progressed recently. Several studies have reported neuropharmacological activities of plant derived terpenes. Farnesol is a sesquiterpene whose most popular source is fruits but the anxiolytic activity for farnesol is still unknown. The present study was conducted on 32 male Swiss Albino mice (8 in each group) to evaluate the neuropharmacological properties of farnesol and its effects on plasma cortisol levels. Farnesol was administered intraperitoneally at single doses of 50 and 100 mg/kg, while diazepam 2 mg/kg was used as standard anxiolytic. Thirty minutes after injections, open field test (OFT), elevated plus maze (EPM), a forced swimming test (FST), and a hot plate test (HPT) were performed for evaluation of anxiety-like behavior, depression and nociception. In OFT, farnesol at the dose of 100 mg/kg led to significant decrease in locomotor activity (P<0.01). In EPM, only farnesol 100 mg/kg led to significant increase in the number of entries to the open arms and the time spent in open arms (P<0.01). Increase in immobility time in FST was seen in farnesol 50 and 100 mg/kg (P<0.001). Farnesol 100 mg/kg exerts significant prolongation in the latency of responses to noxious heat stimuli in HPT. Like diazepam, farnesol decreased plasma levels of cortisol. Results revealed that farnesol had anxiolytic, anti-nociceptive and depressant effects in murine models. The present study provides pharmacological evidence supporting the use of farnesol as a sedative for anxiety disorders.

## Introduction

 Anxiety is the most common behavioral disorder. It is estimated that approximately 400 million people around the world are suffering from anxiety (Lee et al., 2011 ▶). Anxiety is a biological-hormonal response to social and psychological conditions that affect the hypothalamic-pituitary-adrenal (HPA) axis (Flandreau et al., 2011 ▶) followed by an increase in epinephrine concentrations.

 Essential oils derived from plants show a variety of neuropharmacological properties such as anxiolytic (Silva et al., 2007 ▶; De Sousa, 2011 ▶), anti-nociceptive (Melo et al., 2010; Couto et al., 2011 ▶) and anti-convulsant effects (Nóbrega de Almeida et al., 2011 ▶), which are frequently attributed to terpenes (Gomes et al., 2010 ▶). Farnesol, a 15-carbon sesquiterpene (an isoprenoid intermediate of the mevalonate pathway), is produced in cells by the dephosphorylation of farnesyl pyrophosphate, a precursor of squalene generating-sterols and other isoprenoid compounds (Khan and Sultana, 2011 ▶). Fruits such as apricots, peaches, plums and berries are the most popular source of farnesol (Tatman and Mo, 2002 ▶). It could also be found in the essential oils of ambrette seeds, citronella, and chamomile (Duncan and Archer, 2008 ▶). Previous studies have demonstrated anti-oxidative (Khan and Sultana, 2011 ▶) and anti-inflammatory effects of farnesol (Joo and Jetten, 2010 ▶). The neuroprotective effect of farnesol has been reported in lipopolysaccharide induced-neurodegeneration of the cortex and hippocampus of Swiss Albino mice, attributed to the regulation of intrinsic apoptotic cascade due to the anti-oxidant effect of farnesol (Santhanasabapathy and Sudhandiran, 2015 ▶). Anxiolytic effects of farnesol have not yet been reported. Thus, the present study aimed to investigate the effects of farnesol on a classic murine animal model for depression and anxiety-like behavior.

## Materials and Methods

**Chemicals**

 Farnesol was obtained from Sigma-Aldrich (St Louis, MO, USA). The formula is C15H26O with a molecular weight of 222,37 g/mol. According to Sigma, the degree of purity was >97%. Tween 80 (Sigma, USA), diazepam (Kimidaru, Tehran, Iran) and all other chemicals used were of analytical grade and commercially available.

**Animals**

 Adult male Swiss Albino mice (25-35 g) were purchased from the Pasteur Institute of Iran. The animals were acclimatized for approximately one week before the experiment and kept in standard cages with *ad libitum* access to a standard rodent chow and tap water under a controlled room temperature (20°C) with a constant 12 h light/dark cycle. The research protocol was in accordance with the Pasteur Institute of Iran’s Laboratory Animals guidelines.

**Experimental design**

 Thirty two mice were randomly assigned into 4 groups as follows:

CON: Receiving vehicle

FAR 50: Receiving 50 mg/kg farnesol

FAR 100: Receiving 100 mg/kg farnesol

DZP: Receiving diazepam as an anxiolytic drug

 Thirty minutes before the behavioral tests, animals were treated intraperitoneally either with farnesol, saline, or diazepam. To prepare the injections, farnesol was emulsified with 0.2% Tween 80 and administered at doses of 50 and 100 mg/kg, respectively to FAR 50 and FAR 100 groups. The control group received the vehicle (0.2% Tween 80 in normal saline), and the diazepam group received 2 mg/kg diazepam as a standard anxiolytic. The dose of diazepam was chosen as previously described by Melo et al. (2010) ▶.

**Open field test (OFT)**

 The open field device was made of acrylic glass (30 × 30 × 15 cm), divided into 9 squares. For testing purposes, each mouse was placed in the central square and the number of squares crossed with the four paws as well as rearing and grooming behavior were measured for 5 min (Nogueira Neto et al., 2013 ▶).

**The elevated plus maze (EPM) test**

 The EPM test depends on the natural anxiety-related behavior of rodents to stay in shadows, close to walls and to keep away from heights (Bradley et al., 2007 ▶). The apparatus for mice consisted of two open arms (30 × 5 cm^2^) and two closed arms (30 × 5 × 25 cm^3^) on a 5 × 5 cm^2^ central platform (Costa et al., 2014 ▶). The maze was placed as high as 45 cm in a quiet room. Half an hour after treatment the mouse was placed at the center of the plus maze with its face directing to one of the closed arms. Certain parameters such as number of entries to the open arms (NEOA) and the number of entries in the closed arms and time spent at each arm were recorded for 5 minutes. The equipment was washed and disinfected by ethanol 70% and then dried prior to the test, for each mouse.

**Forced swimming test (FST)**

 This test is widely used to evaluate anti-depressant activity in pharmacology (Petit-Demouliere et al., 2005 ▶). To perform this test the animal was set in a 20-cm-deep tank containing fresh water with a temperature of 25°C. Immobility time (floating with only small movements necessary to keep the head above water) was recorded for 5 minutes (Melo et al., 2010).

**Hot plate test (HPT)**

 Mice were placed on a hotplate at 55 ± 1°C and the time when the hind paw was licked or when the mice started jumping was recorded as the index of response latency (Pinheiro et al., 2010 ▶).

**Determination of plasma cortisol concentration**

 Following behavioral tests, blood was collected by cardiocentesis under anesthesia from the mice to measure cortisol concentration. Sera obtained by centrifuging the samples at 4000 rpm for 10 min, and the levels of cortisol were measured by chemiluminescence immunoassays (Immulite^®^, Diagnostic Products Corporation, Los Angeles, USA), according to the manufacturer’s instructions (Purnell et al., 2004 ▶).

**Statistical analysis**

 All results are expressed as mean ± SEM (standard error of mean) values. The data were analyzed by means of analysis of variance (ANOVA) followed by Student-Newman-Keuls’s post-hoc test. Graph Pad Prism software (version 5.0) was used for analysis. P-values of <0.05 were considered as statistically significant.

## Results

**Open ﬁeld test**

*Crossing*

**Fig. 1:**
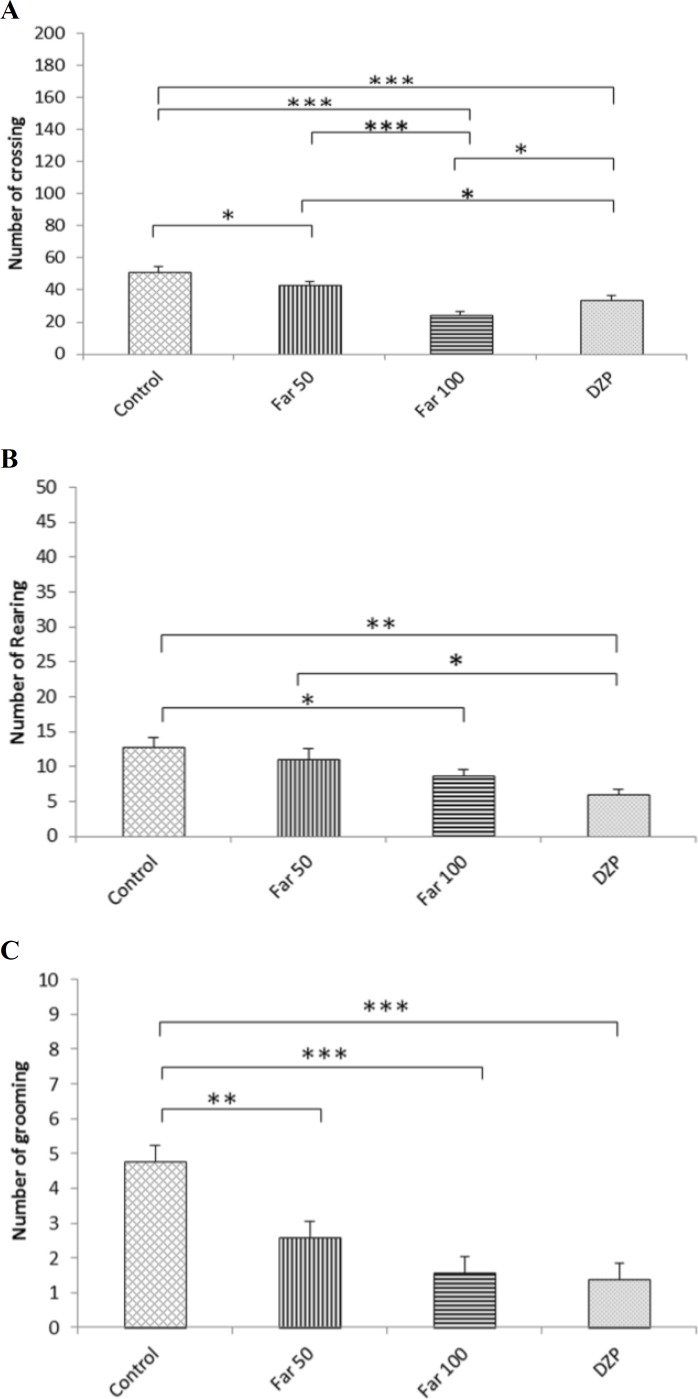
Farnesol effects in mice subjected to the open field test. (A) Number of crossings, (B) Number of grooming, and (C) Number of rearing. Control (vehicle), DZP (diazepam), and Far (farnesol). Each column represents the mean ± SEM (n=8). ^*^ P<0.05, ^**^ P<0.01, and ^***^ P<0.001

 As seen in Fig. 1A, 100 mg/kg farnesol led to a significant decrease in locomotor activity in comparison to the control, the farnesol 50 mg/kg (P<0.001) and the diazepam -treated (P<0.01) mice. A significant difference was found between the FAR 50 and the control group (P<0.05). As expected, animals treated with diazepam showed a significant decrease in crossing compared to the control (P<0.01). The crossing number for diazepam-treated mice and FAR 50 was also statistically different (P<0.05).

*Rearing*

 Only the 100 mg/kg farnesol dose reduced rearing number as compared with the control group (P<0.05). Diazepam-treated mice had a lower number of exploratory rearing in comparison to the control group (P<0.01) and FAR 50 (P<0.05) (Fig. 1B).

*Grooming*

 All treatment groups showed a significant decrease in grooming number in comparison with the control (Fig. 1C).

**Elevated plus maze test**

**Fig. 2:**
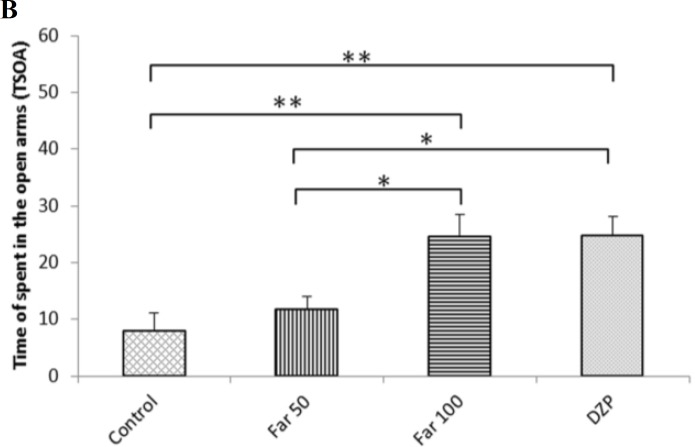
Farnesol effects in mice subjected to elevated plus maze test. (A) Number of entries in open arms, and (B) Time spent in open arms (TSOA). Control (vehicle), DZP (diazepam), and Far (farnesol). Each column represents the mean ± SEM (n=8). ^*^ P<0.05 and ^**^ P<0.01

 The intraperitoneal administration of 100 mg/kg farnesol significantly increased the number of entries in the open arms (Fig. 2A) and the time spent in open arms as compared to the control (P<0.01) (Fig. 2B). These effects were also seen in mice treated with 2 mg/kg diazepam. Farnesol administration (50 mg/kg) increased the number of entries and time spent in open arms when compared with control, although it was not significant (Figs. 2A and B).

**Forced swimming test**

**Fig. 3:**
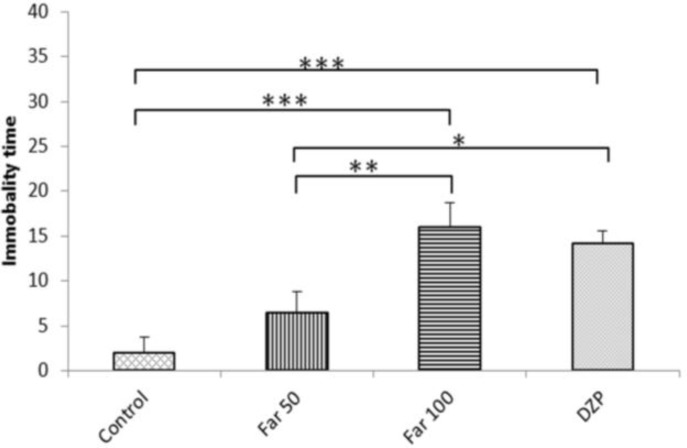
Farnesol effects in mice subjected to the force swimming test. Control (vehicle), DZP (diazepam), and Far (farnesol). Each column represents the mean ± SEM (n=8). ^*^ P<0.05, ^**^ P<0.01, and ^***^ P<0.001

 Significant increase in the immobility time of mice was observed in FAR 100 (P<0.001) and diazepam-treated (P<0.01) mice as compared to the control and FAR 50. Immobility time was longer in FAR 100 mice than the diazepam group (Fig. 3).

**Hot plate test**

**Fig. 4:**
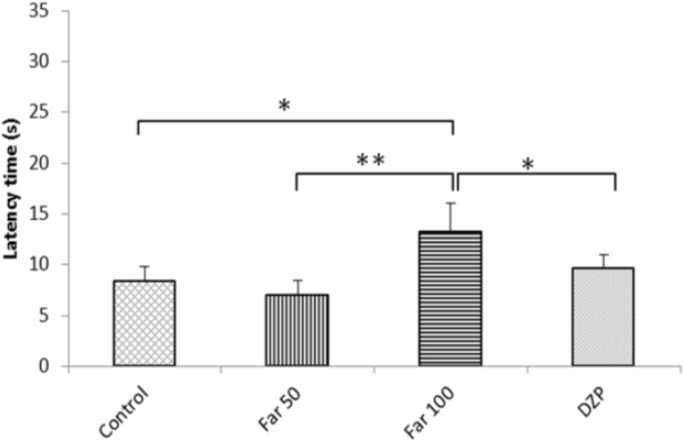
Farnesol effects in mice subjected to hot plate test. Control (vehicle), DZP (diazepam), and Far (farnesol). Each column represents the mean ± SEM (n=8). ^*^ P<0.05 and ^**^ P<0.01

 Administration of 100 mg/kg farnesol resulted in significant prolongation of the latency of response time to acute noxious heat stimuli in comparison to all other groups (P<0.05; Fig. 4).

**Plasma cortisol level**

**Fig. 5:**
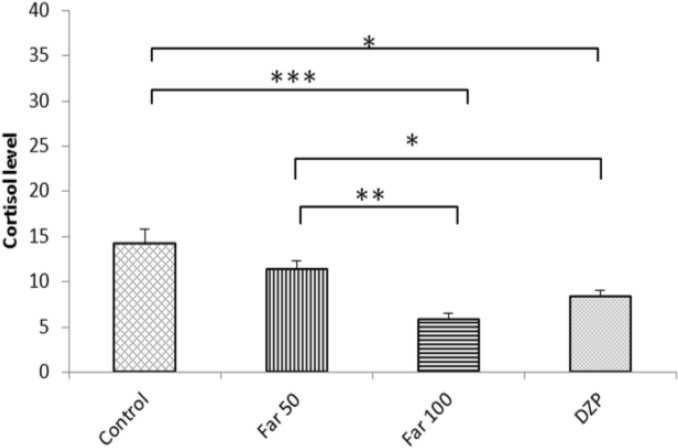
Effects of farnesol on plasma cortisol levels. Control (vehicle), DZP (diazepam), and Far (farnesol). Each column represents the mean ± SEM (n=8). Each column represents the mean ± SEM (n=8). ^*^ P<0.05, ^**^ P<0.01, and ^***^ P<0.001

 Cortisol level was significantly lower in FAR 100 (P<0.001) and diazepam-treated (P<0.01) groups; however, in FAR 50, no significant changes were evident in plasma cortisol levels as compared to the control (Fig. 5).

## Discussion

 In the present study, we investigated the anxiolytic-like, depressant, and anti-nociceptive effects of two different doses of farnesol on behavioral models of central nervous system (CNS) actions (EPM, OFT, FST, and HPT), and compared them with the effects of diazepam. Diazepam is used as a standard anti-anxiety medication as well as a reference compound to evaluate the anxiolytic potential of substances in behavioral pharmacology (Gomes et al., 2010 ▶; Mizushige et al., 2013 ▶).

 Our results demonstrated that farnesol at 50 and 100 mg/kg doses reduces locomotor activity in the OFT. These results are inconsistent with those of other researchers who observed anxiolytic-like action without altering locomotor activities (de Almeida et al., 2012 ▶; Moreira et al., 2014 ▶), On the other hand, **Neto *et al*. (2013)** reported that nerolidol-treated animals showed significantly reduced locomotor activity and number of times grooming and rearing in comparison with control animals, which was in line with observations of the present study. The reduction in the frequency and amplitude of motion may be attributed to the anxiolytic-like activity of farnesol caused by its sedative effect on CNS.

 In EPM, normal mice prefer to stay more in the closed arms because of the fear of open space (Saha et al., 2013 ▶), so compounds that increase the time spent in open arms (TSOA) are considered anxiolytic (Nic Dhonnchadha et al., 2003 ▶). In this study, farnesol, especially the 100 mg/kg dose, induced significant increase in the number of entries and the TSOA as compared to the control animals, which is similar to the effect of diazepam. Our results are in accordance with previous experiments demonstrating anxiolytic-like effects of some monoterpenes such as (+)-limonene epoxide (de Almeida et al., 2012 ▶), linalool oxide (Linck et al., 2010 ▶), 1,4-cineole (Gomes *et al*., 2010), carvacryl acetate (Pires et al., 2013 ▶), phytol (Costa et al., 2014 ▶), carvacrol (Melo et al., 2010) and myrtenol (Moreira et al., 2014 ▶).

 The inhibition on nociception of 100 mg/kg farnesol was evident in our study in HPT. It is noteworthy that acute supraspinal pain model in HPT has been employed over the last six decades to assess the analgesic activity of various drugs in rodents (Casarrubea et al., 2012 ▶). Sulaiman et al. (2009 ▶) investigated the significant dose-dependent anti-nociceptive activity of zerumbone, a natural cyclic sesquiterpene by intraperitoneal route, in acetic acid-induced abdominal writhing test and HPT. Topical application of a preparation containing plant derived substances such as farnesol is patented for use in the prevention and treatment of pain (Guimarães et al., 2014 ▶). In this study, the effectiveness of farnesol in HPT and the significant anti-nociceptive effect, suggest the analgesic effect of this terpene via the central mechanisms (supraspinally) (Sulaiman et al., 2009 ▶). The anti-nociceptive effect of farnesol at 50, 100 and 200 mg/kg has been previously shown in formalin tests and acetic acid induced writhing tests in mice (de Oliveira Júnior et al., 2013 ▶). Farnesol was able to reduce nitric oxide production (Marcuzzi et al., 2010 ▶) which is involved in the development of pain (Sulaiman et al., 2009 ▶). Melo et al. (2010) reported depressant, hypnotic, and anti-nociceptive properties of a monoterpenoid (citronella) in a mouse behavioral model, which was in line with the results obtained using farnesol in the present study.

 An investigation by Costa et al. (2011 ▶) demonstrated the anxiolytic-like effect of a 10 mg/kg dose of essential oils from *Cymbopogon citratus*, without any effect on depression (swimming event in FST) (Costa et al., 2011 ▶). In contrast to these findings, in the present study, farnesol increased immobility time in FST and did not reveal any anti-depressant effect. Farnesol led to a shorter duration of mobility which may reflect the CNS depression-like effect of this compound. In line with the findings of present study, Silva et al. (2007) ▶ reported significant increases in immobility time in FST for isopulegol, a monoterpene alcohol. They also showed that isopulegol exerts depressant and anxiolytic-like effects similar to those observed for farnesol in the present study. The results of EPM, OFT, and FST presented in this study show that farnesol administration, especially with a dose of 100 mg/kg, had anti-anxiety effects like diazepam, inducing sedative action in mice and suggesting possible depression of CNS. However, the exact underlying mechanism remains unclear and needs further examiniation.

 There are reports stating that the anxiolytic properties of essential oils from aromatic herbs are due to the inhibition of the HPA axis hyperactivity (Li et al., 2012 ▶). The anxiolytic effect of bergamot oil and reduction in corticostrone level in rats were a result of a decrease in HPA activity (Saiyudthong and Marsden, 2011 ▶). Li et al. (2012) ▶ reported that propolis essential oil reverses anxiety-like behavior in rats and dramatically decreases the plasma levels of cortisol. They attributed this to the function of the HPA axis. An additional mechanism that could be suggested for the anxiolytic-like effect of farnesol, especially the 100 mg/kg dose, may be through reducing the level of plasma cortisol by affecting the HPA axis, similar to what Dubey et al. (2015) ▶ demonstrated for chromium picolinate (Dubey et al., 2015 ▶). Nevertheless, more studies should be carried out to reveal the underlying mechanisms of neuropharma-cological properties of farnesol.

 The present study provides pharmacological support for the possible use of farnesol as a sedative for the relief of anxiety disorders. The results suggest that farnesol possesses potential anxiolytic-like actions and induces depression and alterations in locomotor activity. Further studies are needed, however, to understand the underlying mechanism of these effects.
